# Effects of the Mutant TP53 Reactivator APR-246 on Therapeutic Sensitivity of Pancreatic Cancer Cells in the Presence and Absence of WT-TP53

**DOI:** 10.3390/cells11050794

**Published:** 2022-02-24

**Authors:** Stephen L. Abrams, Przemysław Duda, Shaw M. Akula, Linda S. Steelman, Matilde L. Follo, Lucio Cocco, Stefano Ratti, Alberto M. Martelli, Giuseppe Montalto, Maria Rita Emma, Melchiorre Cervello, Dariusz Rakus, Agnieszka Gizak, James A. McCubrey

**Affiliations:** 1 Department of Microbiology and Immunology, Brody School of Medicine, East Carolina University, Greenville, NC 27858, USA; abramss@ecu.edu (S.L.A.); akulas@ecu.edu (S.M.A.); lssteelman@gmail.com (L.S.S.); 2 Department of Molecular Physiology and Neurobiology, University of Wrocław, 50-335 Wrocław, Poland; przemyslaw.duda@uwr.edu.pl (P.D.); dariusz.rakus@uwr.edu.pl (D.R.); agnieszka.gizak@uwr.edu.pl (A.G.); 3 Dipartimento di Scienze Biomediche e Neuromotorie, Università di Bologna, 40139 Bologna, Italy; matilde.follo@unibo.it (M.L.F.); lucio.cocco@unibo.it (L.C.); stefano.ratti@unibo.it (S.R.); alberto.martelli@unibo.it (A.M.M.); 4 Department of Health Promotion, Maternal and Child Care, Internal Medicine and Medical Specialties, University of Palermo, 90127 Palermo, Italy; giuseppe.montalto@unipa.it; 5 Institute for Biomedical Research and Innovation, National Research Council (CNR), 90146 Palermo, Italy; mariarita.emma@irib.cnr.it (M.R.E.); melchiorre.cervello@irib.cnr.it (M.C.)

**Keywords:** TP53, mutant TP53 reactivators, nutlin-3a, targeted therapy, PDAC

## Abstract

The TP53 tumor suppressor is mutated in ~75% of pancreatic cancers. The mutant TP53 protein in pancreatic ductal adenocarcinomas (PDAC) promotes tumor growth and metastasis. Attempts have been made to develop molecules that restore at least some of the properties of wild-type (WT) TP53. APR-246 is one such molecule, and it is referred to as a mutant TP53 reactivator. To understand the potential of APR-246 to sensitize PDAC cells to chemotherapy, we introduced a vector encoding WT-TP53 into two PDAC cell lines, one lacking the expression of TP53 (PANC-28) and one with a gain-of-function (GOF) mutant TP53 (MIA-PaCa-2). APR-246 increased drug sensitivity in the cells containing either a WT or mutant TP53 protein with GOF activity, but not in cells that lacked TP53. The introduction of WT-T53 into PANC-28 cells increased their sensitivity to the TP53 reactivator, chemotherapeutic drugs, and signal transduction inhibitors. The addition of WT-TP53 to PDAC cells with GOF TP53 also increased their sensitivity to the drugs and therapeutics, indicating that APR-246 could function in cells with WT-TP53 and GOF TP53. These results highlight the importance of knowledge of the type of TP53 mutation that is present in cancer patients before the administration of drugs which function through the reactivation of TP53.

## 1. Introduction

Pancreatic cancer accounts for the second highest number of cancer deaths [1,2]. The 5-year survival rate for pancreatic cancer is very low. Approximately 85% of pancreatic cancers are pancreatic ductal adenocarcinomas (PDAC).

The removal of the diseased portion of the pancreas is a treatment for PDAC [3]. Unfortunately, the tumor often reappears and may have metastasized to other organs, which makes effective treatment more difficult, if not impossible.

Chemotherapy has been used for decades to treat various cancer patients. Some cancers are susceptible to chemotherapy and success occurs. Chemotherapy has also been used to treat PDAC patients; however, it is usually a palliative as opposed to curative approach, and only some patients respond [4,5].

Many genes have been implicated in PDAC [6,7]. Two of the most familiar mutated genes are the *KRAS* oncogene, which encodes an oncoprotein that is constitutively active in PDAC cells [8], and the *TP53* tumor suppressor gene that encodes a tumor suppressor oncoprotein that has altered activity in the cells [9]. Some *TP53* mutations result in novel activities for the TP53 protein. These mutations are called gain-of-function (GOF) mutations. Another type of TP53 mutation in PDAC results in deletion (either partial or full deletion) in one or both alleles, and the full length TP53 protein may not be expressed. Certain mutant TP53 oncoproteins will activate oncogenic Ras signaling [10]. Despite our understanding of the key genes implicated in PDAC, therapy remains limited. Thus, additional, more effective approaches to treat PDAC are needed.

An approach to inhibit the effects of mutant GOF TP53 is the isolation of small molecule TP53 activators (reactivators) that interact with the mutant TP53 protein and restore some of its tumor suppressor activity. An example of such reactivators is APR-246 [11,12,13,14,15]. It is also known as PRIMA-1Met, and clinically as Eprenetapopt [14,15]. Recently, Eprenetapopt received Breakthrough Therapy, Orphan Drug, and Fast Track designations from the FDA for the treatment of patients with myelodysplastic syndrome (MDS) and acute myeloid leukemia (AML) who have mutant TP53, in combination with the nucleoside analog azacitidine [15].

**Figure 1:**
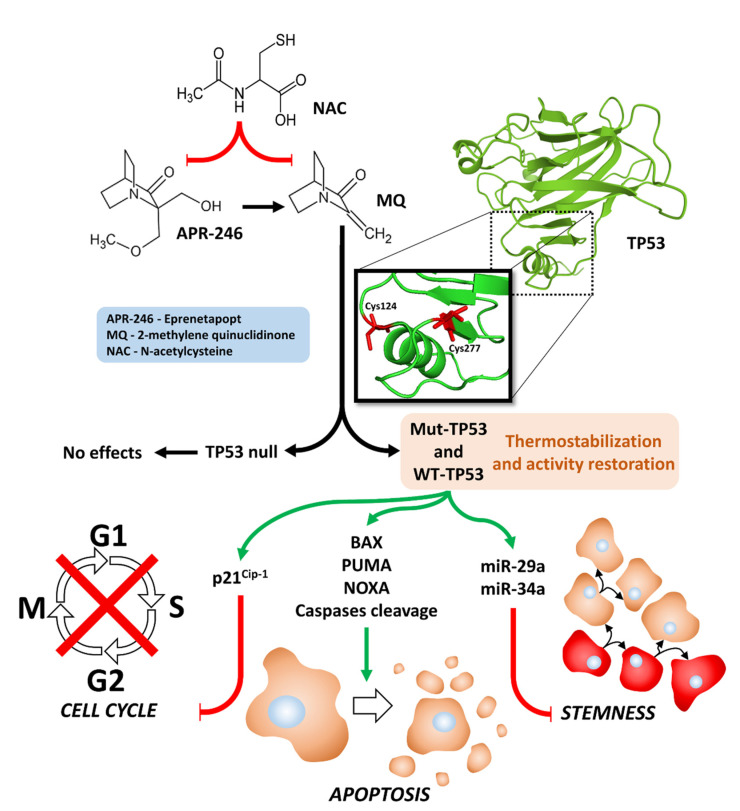
Illustration of effects of APR-246 on mutant and WT TP53 activities, inhibition of cell cycle progression, induction of apoptosis, induction of miRs, and inhibition of stemness.

APR-246 is a prodrug, and it is converted into reactive electrophile 2-methylene quinuclidinone (MQ) to become the active form, which is a Michael acceptor [16]. MQ binds critical cysteines in the core binding domain of TP53 (C277 and C124) and changes its conformation, which results in the thermostabilization of TP53. This can result in the reactivation of some of TP53 activities [17]. A diagram of these interactions is presented in Figure 1. Mutant TP53 “reactivators” may function as chaperones and may also bind related TP63 and TP73 proteins [18]. They stabilize the proteins and maintain the correctly folded protein conformation [18]. APR-246 induces reactive oxygen species (ROS) production [19,20]. ROS could have multiple effects on the cells and alter the structure of the mutant TP53 protein, which allows the mutant protein to have some of the growth-regulatory effects present in WT TP53 [21,22].

Thus, APR-246 is an interesting and important compound that has shown the ability to reactivate important aspects of the mutant TP53 tumor suppressor protein. In the following study, we have examined the effects of APR-246 on PDAC cells which have GOF TP53 proteins or lack TP53 (TP53 null), and the same cell lines with introduced WT-TP53.

## 2. Materials and Methods

### 2.1. Cell Lines and Culture

The MIA-PaCa-2 PDAC cell line (ATCC CRM-CRL-1420) was obtained from the American Type Culture Collection (ATCC) (Manassas, VA, USA). The cells were recovered from a 65-year-old Caucasian male PDAC patient [23]. MIA-PaCa-2 cells have an activating mutation in the *KRAS* gene. Both *KRAS* alleles have codon 12 mutations (GGT → GAT) and GOF TP53 mutations (R248W) [23,24]. This is the most common TP53 mutation in human cancer [24]. The MDA-PANC-28 cell line was obtained from Dr. Shrikanth A. G. Reddy, MD, Anderson Cancer Center (Houston, TX, USA). The MDA-PANC-28 cells were obtained from a female 69-year-old PDAC patient and established into a cell line at the MD Anderson Cancer Center [25,26]. The MDA-PANC-28 cell line is frequently abbreviated to PANC-28. PANC-28 have an activating mutation in the *KRAS* gene. PANC-28 cells are heterozygous for KRas (protein: p. Gly12Asp, nucleotide (c.35G > A)). No detectable TP53 protein was reported in PANC-28 [26].

The cell culture medium consisted of 5% (*v/v*) heat-inactivated fetal bovine serum (FBS) (CellGrow-Mediatech, Herndon, VA, USA), 2 mM L-glutamine (Invitrogen, Carlsbad, CA, USA), 100 μg/mL streptomycin (Invitrogen, Carlsbad, CA, USA and Thermo Fisher, Waltham, MA, USA), and 100 units/L penicillin G (Invitrogen) in Dulbecco’s Modified Eagles Medium (DMEM) (Invitrogen).

The PA317 retroviral packaging cell line was transfected plasmid DNA with either the empty retroviral vector pLXSN or pCMV/p53WT inserted into pLXSN. Stable pools of infected cells were selected in a medium containing 2 mg/mL geneticin (G418). After 2 weeks, the selective medium was removed, and the medium lacking geneticin was added to generate a viral pool of stable transfectants. Supernatants were prepared and sterilized through a 0.45 µM Acrodisk syringe filter (Pall Corporation, Port Washington, NY, USA). PDAC cells were infected with the respective retroviral supernatants in the presence of 10 µg/mL polybrene from Sigma-Aldrich (Saint Louis, MO, USA) for 2 h, and then the medium was removed, and fresh selection medium was added, to generate pools of stable transfectants. The PDAC cell lines were infected with the either the retrovirus encoding WT-TP53 or the pLXSN empty vector, as described previously [27,28].

### 2.2. Chemotherapeutic Drugs and Small Molecule Signal Transduction Inhibitors

Chemotherapeutic drugs and signal transduction inhibitors were obtained from either Sigma-Aldrich (Saint Louis, MO, USA) or Selleck Chemicals (Houston, TX, USA). The following chemotherapeutic drugs and signal transduction inhibitors were used in this study: APR-246, a mutant TP53 reactivator (Selleck Chemicals), 5-fluorouracil (5FU), a nucleoside analogue (Sigma-Aldrich, Saint Louis, MO, USA), doxorubicin (Dox), a topoisomerase II inhibitor (Sigma Aldrich, Saint Louis, MO, USA), gilteritinib, an ALK/AXL/FLT3 inhibitor (Selleck Chemicals), ABT-737, a BCL2/BCXL inhibitor (Selleck Chemicals).

APR-246 (Eprenetapopt) is used to treat certain leukemias with TP53 mutations (acute myeloid leukemias, myelodysplastic syndromes) and potentially some other cancers. In addition, 5FU (adrucil, Carac, Efudex) is used to treat various types of cancer, including PDAC. Doxorubicin (adriamycin) is used to treat various cancers, including leukemia and breast cancer. Gilteritinib (Xospata) is used to treat leukemias and potentially certain other cancers. ABT-737 is a prototype of ABT-199 (venetoclax, which does not inhibit BCLXL), and is used to treat some leukemias, such as chronic lymphocytic leukemia.

### 2.3. Introduction of Either WT-TP53 or a Control Plasmid into MIA-PaCa-2 and PANC-28 Cells

Plasmid DNA encoding cDNA encoding WT-TP53 subcloned in the pLXSN retroviral vector was generously provided by Dr. Moshe Oren [27,28] (Rehovot, Israel). The WT-TP53 construct also encodes the resistance to geneticin (G418). Plasmid pLXSN encodes resistance to G418 and was generously provided by A. Dusty Miller (Fred Hutchinson Cancer Center, Seattle, Washington, DC, USA) [29]. MIA-PaCa-2 and PANC-28 cells containing either WT-TP53 or pLXSN have been previously described [30,31,32].

### 2.4. Cell Proliferation Assays in the Presence of Chemotherapeutic Drugs and Signal Transduction Inhibitors

MIA-PaCa-2 + pLXSN, MIA-PaCa-2 + WT-TP53, PANC-28 + pLXSN, and PANC-28 + WT-TP53 cells were seeded into 96-well cell culture plates (BD Biosciences, Bedford, MA, USA) at a density of 5000 cells/well in 100 μL of phenol red free RPMI-1640 containing 1% FBS. Cell culture plates were incubated for one day to allow cells to adhere to the bottom of each well [32]. The treatment medium was prepared by performing ten two-fold serial dilutions to create a range of eleven concentrations of the different drugs, signal transduction inhibitors, and nutraceuticals. After 72 h of treatment (four days after seeding), the tetrazolium-based cell growth/viability assay was performed. The amount of 3-(4,5-dimethylthiazol-2-yl)-2,5-diphenyl-2*H*-tetrazolium bromide (MTT) (Sigma-Aldrich, Saint Louis, MO, USA) reduction in each well was quantified by dissolving the formazan crystals in 200 μL of dimethyl sulfoxide (DMSO) and reading the absorbance at 570 nM with a FL600 microplate fluorescence reader (Bio-Tek Instruments; Winooski, VT, USA). Control plates were read on day one and day four after seeding to provide a baseline for cell growth. The mean and corresponding standard deviation of normalized adjusted absorbance was calculated from three replicate wells for each drug concentration. The inhibitory concentration of 50% (IC_50_) is defined, in this context, as the concentration of the drug that causes MIA-PaCa-2 cells to proliferate at a rate that is half as rapid as cells incubated in the absence of the drug.

### 2.5. Clonogenicity Assays

MIA-PaCa-2 + pLXSN, MIA-PaCa-2 + WT-TP53, PANC-28 + pLXSN, and PANC-28 + WT-TP53 cells were collected and seeded in six-well cell culture plates at a density of 500 cells/well in 2 mL of DMEM + 5% FBS for each well (three replicate wells for each condition), as described [33]. Cell culture plates were incubated for one day to allow cells to adhere to the bottom of each plate. Then, 24 h after seeding, plates were treated with different concentrations of APR-246 and, in some cases, low doses of either 5FU or doxorubicin in 2 mL of DMEM + 5% FBS for each well, and were incubated for three weeks at 37 °C. Cells were provided with fresh treatment-containing media every four days. Cells were rinsed with PBS at the end of the three-week treatment period. Fixed cells were incubated in Giemsa stain (Sigma) for five minutes at room temperature. Stained cells were rinsed with water and then dried. Colonies consisted of at least 50 cells and the number of colonies present in each well was counted. The mean number of colonies and corresponding standard deviation was calculated from three replicate wells for each condition. Colonies were normalized to untreated controls which did not receive any drugs. Statistical significance was calculated using the GraphPad QuickCalcs software (San Diego, CA, USA) using an unpaired *t* test with a 95% confidence interval.

## 3. Results

### 3.1. Effects of APR-246 on Clonogenicity of MIA-PaCa-2 and PANC-28 Cells Containing and Lacking WT-TP53

**Figure 2:**
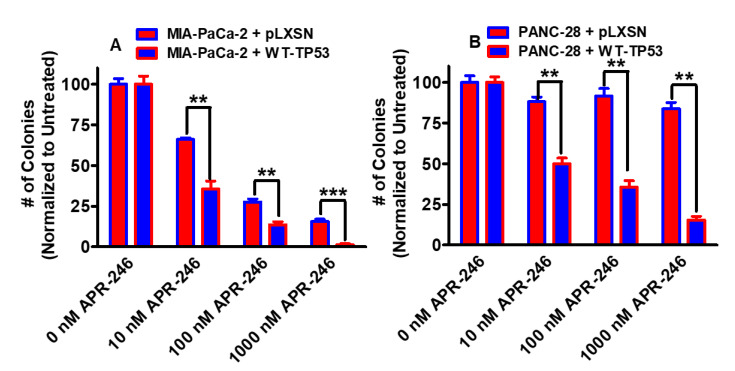
Effects of APR-246 on clonogenicity of PDAC cells containing and lacking WT-TP53 or mutant GOF TP53. Panel (**A**) MIA-PaCa-2 + pLXSN cells (red bars), MIA-PaCa-2 + WT-TP53 cells (blue bars). Panel (**B**) PANC-28 + pLXSN cells (red bars), PANC-28 + WT-TP53 cells (blue bars). Cells were examined for their abilities to form colonies in the presence of different concentrations of APR-246. These experiments were repeated, and similar results were obtained. Statistical analyses were performed with Student’s *t* test on the means and standard deviations of various treatment groups. *** = *p* < 0.0001, and ** = *p* < 0.005.

The effects of the mutant TP53 APR-246 reactivator APR-246 on clonogenicity were examined in two pancreatic cell lines: MIA-PaCa-2, which contains GOF mutant TP53 on both alleles [23,24], and PANC-28, which does not produce detectable TP53 [25,26]. As observed in Figure 2, APR-246 inhibited clonogenicity in MIA-PaCa-2 cells in the absence and presence of WT-TP53 (Panel A). APR-246 had less effects on PANC-28 + pLXSN cells, which lacked WT-TP53 (Panel B). In contrast, APR-246 did inhibit the colony formation in PANC-28 + WT-TP53 cells in a dose-dependent fashion.

### 3.2. Effects of APR-246 on Cell Growth in MIA-PaCa-2 and PANC-28 Cells Containing and Lacking WT-TP53

**Figure 3:**
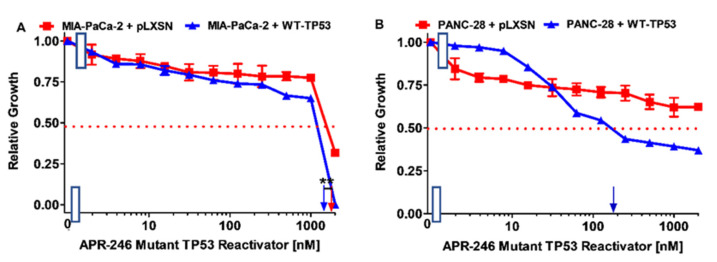
Effects of APR-246 on growth of PDAC cells containing and lacking WT-TP53 and/or mutant GOF TP53. Panel (**A**) MIA-PaCa-2 + pLXSN cells (red squares) and MIA-PaCa-2 + WT-TP53 cells (blue triangles). Panel (**B**) PANC-28 + pLXSN cells (red squares) and MIA-PaCa-2 + WT-TP53 cells (blue triangles). These experiments were repeated, and similar results were obtained. Statistical analyses were performed with Student’s *t* test on the means and standard deviations of various treatment groups. ** = *p* < 0.005.

We next examined the effect of APR-246 on cell growth with an MTT assays, as we needed to titrate APR-246 to determine a suitable concentration for further studies with APR-246 in combination with chemotherapeutic drugs or signal transduction inhibitors. When MIA-PaCa-2 + pLXSN and MIA-PaCa-2 + WT-TP53 were treated with APR-246, the IC_50_ values were approximately 1800 nM and 1500 nM, respectively (Figure 3, Panel A). Thus, the introduction of a WT-TP53 gene increased the sensitivity to APR-246 about 1.2-fold compared to MIA-PaCa-2 cells which lacked WT-TP53.

When PANC-28 + pLXSN and PANC-28 + WT-TP53 were treated with APR-246, the IC_50_ values were >2000 nM and approximately 200 nM, respectively (Figure 3, Panel B). Thus, the introduction of a WT-TP53 gene increased the sensitivity to APR-246 >10-fold compared to MIA-PaCa-2 cells which lacked WT-TP53. Thus, at these concentrations, APR-246 did not have significant effects on PANC-28 cells which lacked the expression of WT-TP53. In addition, these experiments indicated that the restoration of WT TP53 activity sensitized the PANC-28 cells to the APR-246 compound.

### 3.3. Abilities of a Low Dose of APR-246 to Decrease the IC_50_ Values of Chemotherapeutic Drugs and Signal Transduction Inhibitors of MIA-PaCa-2 Cells Containing and Lacking WT-TP53

The abilities of a low dose of APR-246 to reduce the IC_50_ values of chemotherapeutic drugs and signal transduction inhibitors were determined in MIA-PaCa-2 + pLXSN and MIA-PaCa-2 + WT-TP53 cells.

**Figure 4:**
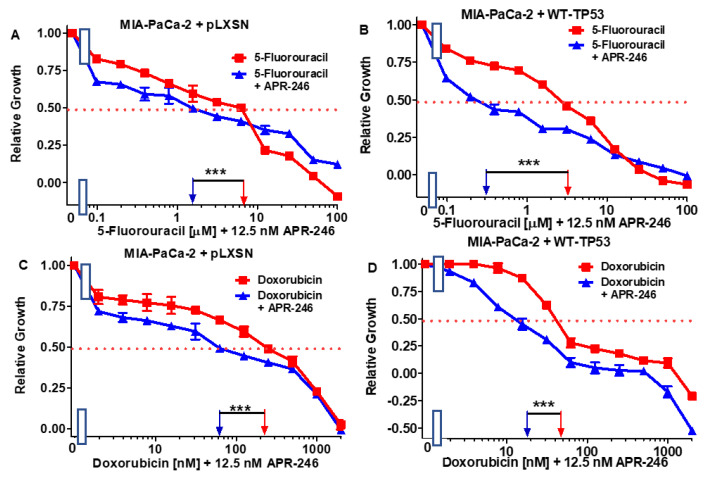
Effects of combining a low concentration of 12.5 nM APR-246 on the IC_50_ values of 5FU and doxorubicin in MIA-PaCa-2 + pLXSN and MIA-PaCa-2 + WT-TP53 Cells. Panel (**A**) MIA-PaCa-2 + pLXSN cells treated with different concentrations of 5FU (red squares) and MIA-PaCa-2 + pLXSN cells treated with different concentrations of 5FU and a low dose of 12.5 nM APR-246 (blue triangles). Panel (**B**) MIA-PaCa-2 + WT-TP53 cells treated with different concentration of 5FU (red squares) and MIA-PaCa-2 + WT-TP53 cells treated with different concentrations of 5FU and a low dose of 12.5 nM APR-246 (blue triangles). Panel (**C**) MIA-PaCa-2 + pLXSN cells treated with different concentrations of doxorubicin (red squares) and MIA-PaCa-2 + pLXSN cells treated with different concentrations of doxorubicin and a low dose of 12.5 nM APR-246 (blue triangles). Panel (**D**) MIA-PaCa-2 + WT-TP53 cells treated with different concentration of doxorubicin (red squares) and MIA-PaCa-2 + WT-TP53 cells treated with different concentrations of doxorubicin and a low dose of 12.5 nM APR-246 (blue triangles). *** = *p* < 0.0001.

**Figure 5:**
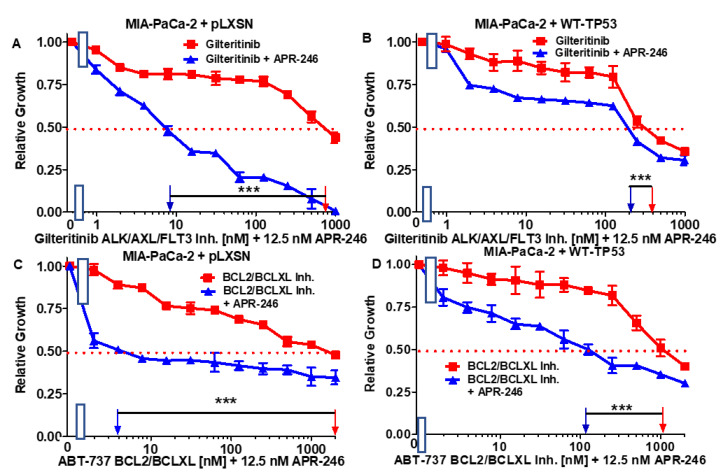
Effects of combining a low concentration of 12.5 nM APR-246 on the IC_50_ values of gilteritinib and ABT-737 in MIA-PaCa-2 + pLXSN and MIA-PaCa-2 + WT-TP53 cells. Panel (**A**) MIA-PaCa-2 + pLXSN cells treated with different concentrations of gilteritinib (red squares) and MIA-PaCa-2 + pLXSN cells treated with different concentrations of gilteritinib and a low dose of 12.5 nM APR-246 (blue triangles). Panel (**B**) MIA-PaCa-2 + WT-TP53 cells treated with different concentrations of gilteritinib (red squares) and MIA-PaCa-2 + WT-TP53 cells treated with different concentrations of gilteritinib and a low dose of 12.5 nM APR-246 (blue triangles). Panel (**C**) MIA-PaCa-2 + pLXSN cells treated with different concentrations of ABT-737 (red squares) and MIA-PaCa-2 + pLXSN cells treated with different concentrations of gilteritinib and a low dose of 12.5 nM APR-246 (blue triangles). Panel (**D**) MIA-PaCa-2 + WT-TP53 cells treated with different concentrations of ABT-737 (red squares) and MIA-PaCa-2 + WT-TP53 cells treated with different concentrations of ABT-737 and a low dose of 12.5 nM APR-246 (blue triangles). *** = *p* < 0.0001.

We tested 5FU (a nucleoside analog) [34], doxorubicin (a topoisomerase inhibitor) [35], gilteritinib (an ALK/AXL/FLT3 inhibitor) [36], and a BCL2/BCXL inhibitor (an ABT-737 inhibitor) [37]. The results of this experiment are presented in Figure 4 and Figure 5 and summarized in Table 1.

A combination of one of the drugs with a low dose of APR-246 reduced the IC_50_ for the drugs both in MIA-PaCa-2 + pLXSN and in MIA-PaCa-2 + WT-TP53 cells. The low dose of 12.5 nM APR-246 was determined by titration experiments, as presented in Figure 2 in the clonogenicity assays, with MIA-PaCa-2 + pLXSN, MIA-PaCa-2 + WT-TP53, and PANC-28 + WT-TP53 cells. Doses of 10 nM APR-246 suppressed clonogenicity by approximately 50%. The most effective was the combination of APR-246 with ABT-737 in MIA-PaCa-2 + pLXSN (500-fold reduction of the IC_50_).

### 3.4. Abilities of Low Doses of 5FU or Doxorubicin to Increase the Cytotoxicity of APR-246 and Decrease Clonogenicity of MIA-PaCa-2 Cells Containing and Lacking WT-TP53

**Figure 6:**
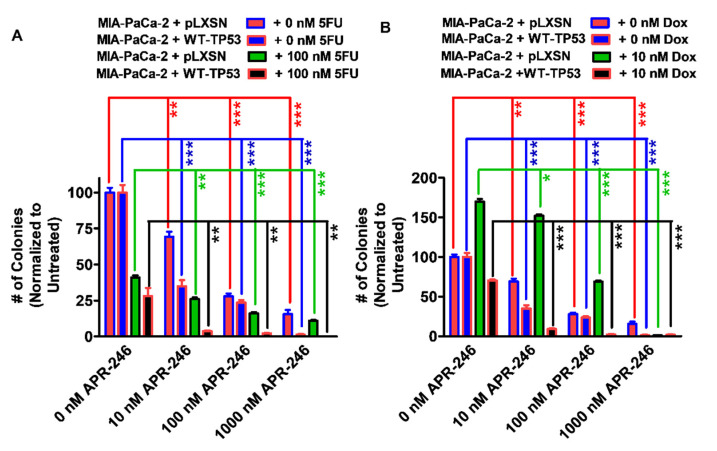
Effects of APR-246 on clonogenicity of MIA-PaCa-2 + pLXSN and MIA-PaCa-2 + WT-TP53 cells in the presence and absence of low doses of 5FU or doxorubicin. The effects of ABR-246 on clonogenicity in the presence of low doses of 5FU (Panel (**A**)) or doxorubicin (Panel (**B**)) were examined, with MIA-PaCa-2 + pLXSN in the absence of either 5FU or doxorubicin (red bars), MIA-PaCa-2 + WT-TP53 in the absence of either 5FU or doxorubicin (blue bars), MIA-PaCa-2 + pLXSN in the presence of APR-246 and 5FU or doxorubicin (green bars), and MIA-PaCa-2 + WT-TP53 in the presence of APR-246 with either 5FU or doxorubicin (black bars). In each condition, the cells were plated in 3 wells of a 6–well plate, and cells in each panel were all examined at the same time period. *** = *p* < 0.0001, ** *p* < 0.005, and * *p* < 0.05.

To ascertain whether the results observed with APR-246 and chemotherapeutic drugs in smaller cultures would also exhibit similar effects on larger cultures, the clonogenicity of the cells containing and lacking WT-TP53 in APR-246 were determined with different concentrations of APR-246 and low doses of 5FU or doxorubicin (Figure 6). APR-246 inhibited the clonogenicity of MIA-PaCa-2 + pLXSN and MIA-PaCa-2 + WT-TP53 cells in a dose-dependent fashion. Furthermore, the addition of a low dose of 5FU resulted in the increased suppression of growth (Figure 6, Panel A). The effects of APR-246 were greater when the MIA-PaCa-2 + WT-TP53 cells were treated with higher concentrations of APR-246 and 5FU than in MIA-PaCa-2 + pLXSN cells.

Interestingly, a low dose of doxorubicin stimulated the clonogenicity of MIA-PaCa-2 + pLXSN but not MIA-PaCa-2 + WT-TP53 cells (Figure 6, Panel B). The stimulation of colony formation in MIA-PaCa-2 + pLXSN by the low dose of doxorubicin was suppressed by high doses of APR-246. Previously, we determined that doxorubicin treatment would induce TP53 accumulation in hematopoietic, breast, and prostate cancer cell lines [38,39,40].

### 3.5. Abilities of a Low Dose of APR-246 to Decrease the IC_50_ Values of Chemotherapeutic Drugs and Signal Transduction Inhibitors of PANC-28 Cells Containing and Lacking WT-TP53

**Figure 7:**
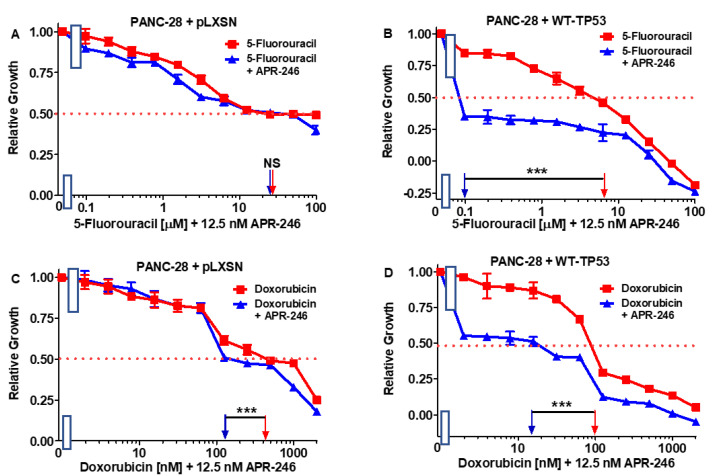
Effects of combining a constant concentration of 12.5 nM APR-246 on the IC_50_ value of 5FU and doxorubicin in PANC-28 + pLXSN and PANC-28 + WT-TP53 cells. Panel (**A**) PANC-28 + pLXSN cells treated with different concentrations of 5FU (red squares) and PANC-28 + pLXSN cells treated with different concentrations of 5FU and a low dose of 12.5 nM APR-246 (blue triangles). Panel (**B**) PANC-28 + WT-TP53 cells treated with different concentrations of 5FU (red squares) and PANC-28 + WT-TP53 cells treated with different concentrations of 5FU and a low dose of 12.5 nM APR-246 (blue triangles). Panel (**C**) PANC-28 + pLXSN cells treated with different concentrations of doxorubicin (red squares) and PANC-28 + pLXSN cells treated with different concentrations of doxorubicin and a low dose of 12.5 nM APR-246 (blue triangles). Panel (**D**) PANC-28 + WT-TP53 cells treated with different concentrations of doxorubicin (red squares) and PANC-28 + WT-TP53 cells treated with different concentrations of doxorubicin and a low dose of 12.5 nM APR-246 (blue triangles). *** = *p* < 0.0001, NS = not statistically significant.

**Figure 8:**
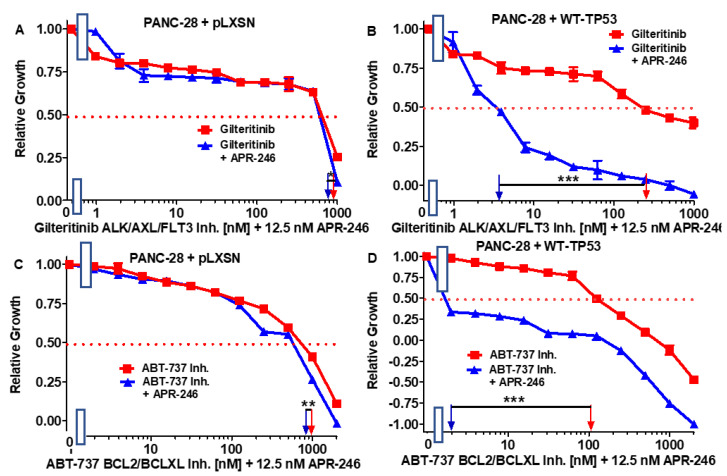
Effects of combining a constant concentration of 12.5 nM APR-246 on the IC_50_ values of gilteritinib and ABT-737 in PANC-28 + pLXSN and PANC-28 + WT-TP53 cells. Panel (**A**) PANC-28 + pLXSN cells treated with different concentrations of gilteritinib (red squares) and PANC-28 + pLXSN cells treated with different concentrations of gilteritinib and a low dose of 12.5 nM APR-246 (blue triangles). Panel (**B**) PANC-28 + WT-TP53 cells treated with different concentrations of gilteritinib (red squares) and PANC-28 + WT-TP53 cells treated with different concentrations of gilteritinib and a constant dose of 12.5 nM APR-246 (blue triangles). Panel (**C**) PANC-28 + pLXSN cells treated with different concentrations of ABT-737 (red squares) and PANC-28 + pLXSN cells treated with different concentrations of ABT-737 and a low dose of 12.5 nM APR-246 (blue triangles). Panel (**D**) PANC-28 + WT-TP53 cells treated with different concentrations of ABT-737 (red squares) and PANC-28 + WT-TP53 cells treated with different concentrations of ABT-737 and a low dose of 12.5 nM APR-246 (blue triangles). *** = *p* < 0.0001, ** *p* < 0.005 and * = *p* < 0.05.

We also tested the same chemotherapeutic drugs and signal transduction inhibitors on the PANC-28 cell line. The results of these experiments are presented in Figure 7 and Figure 8 and summarized in Table 2.

In contrast to the results observed with MIA-PaCa-2 + pLXSN cells, which have GOF mutant TP53 alleles, a low dose of APR-246 did not dramatically change the sensitivity to the various drugs and signal transduction inhibitors in PANC-28 + pLXSN cells, which lack detectable TP53 protein expression. Although there were some statistically differences observed in some cases upon the addition of APR-246 to these cells, the differences were less than 1.3-fold. In turn, when WT TP53 was introduced into PANC-28 cells, they became sensitive to the APR-246. A combination of one of the tested drugs and inhibitors with a low dose of APR-246 resulted in a 7- to 500-fold decrease in their IC_50_ values in the PANC-28 + WT-TP53 cells.

### 3.6. Abilities of a Low Dose of Either 5FU or Doxorubicin to Increase the Cytotoxicity of APR-246 in PANC-28 Cells Containing and Lacking WT-TP53

**Figure 9:**
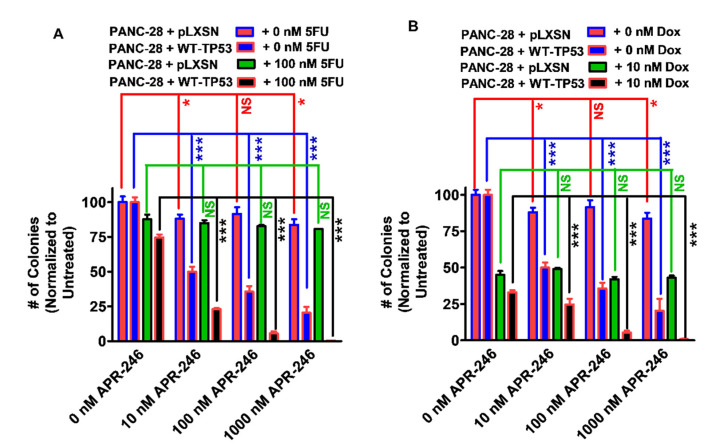
Effects of APR-246 on clonogenicity of PANC-28 + pLXSN and PANC-28 + WT-TP53 cells in the presence and absence low doses of 5FU or doxorubicin. The effects of ABR-246 on clonogenicity in the presence of low doses of 5FU (Panel (**A**)) or doxorubicin (Panel B) were examined, with PANC-28 + pLXSN in the absence of APR-246 (solid red bars), PANC-28 + WT-TP53 in the absence of APR-246 (solid blue bars), PANC-28 + pLXSN in the presence of APR-246 and 5FU (Panel (**A**)) or doxorubicin (Panel (**B**)) (solid green bars), PANC-28 + WT-TP53 in the presence of APR-246 with either 5FU (Panel (**A**)) or doxorubicin (Panel (**B**)) (solid black bars). In each condition, the cells were plated in 3 wells of a 6–well plate, and cells in each panel were all examined at the same time period. *** = *p* < 0.0001, * *p* < 0.05, and NS = not statistically significant.

To ascertain whether the results observed with APR-246 and chemotherapeutic drugs in smaller cultures would also exhibit similar effects on larger cultures, the clonogenicity values of the PANC-28 cells containing and lacking WT-TP53 in APR-246 were determined with different concentrations of APR-246 and low concentrations of either 5FU or doxorubicin (Figure 9, Panels A and B). APR-246 inhibited the clonogenicity of PANC-28 + WT-TP53 cells in a dose-dependent fashion. Furthermore, the addition of a suboptimal concentration of 5FU resulted in the increased suppression of growth in PANC-28 + WT-TP53 cells treated with APR-246. In contrast, APR-246 had less effects on PANC-28 + pLXSN cells. The addition of a low dose of 5FU did not increase the effects of APR-246 in these cells (Panel A). Likewise, the addition of a low dose of doxorubicin resulted in the increased suppression of clonogenicity in PANC-28 + WT-TP53 cells but not in PANC-28 + pLXSN cells (Panel B).

## 4. Discussion

The TP53 oncogene is one of the most frequently mutated genes in human cancer. GOF mutations at TP53 can result in different transcriptional programs which alter cell growth and promote malignant transformation. GOF TP53 mutants (mut-TP53) can repress TP73/nuclear factor Y (NF-Y) transcription factor complex formation on the platelet-derived growth factor receptor-β promoter region. NF-Y can then induce PDGFR-β expression, which is stimulated by the production of autocrine PDGF, which drives PDAC metastasis [41]. Normally TP73 binds NF-Y, which suppresses PDGFR-β expression. PDGFR-β expression is also suppressed in TP53-null cells due to TP73 binding NF-Y. In the presence of increased PDGFR-β expression, mut-TP53 can increase the extent of fibrosis and reduce the infiltration of cytotoxic CD8+ lymphocytes, which contributes to metastasis [42]. Mut-TP533 GOF mutations may promote a fibrotic tumor microenvironment, which suppresses the immune system to eliminate the PDAC. This leads to a poor PDAC prognosis by promoting a more strenuous fibrotic tumor microenvironment [42].

In a later study by a different research group, it was determined that mut-TP53 and mut-KRas cooperate with the ADP ribosylation factor 6 (ARF6) and AMAP1 (ARF6/AMAP1) pathways [43]. AMAP1 is a downstream substrate of ARF6 and is important in invasion, metastasis, and endosome recycling [44]. Mut-KRas promotes eukaryotic initiation factor-4A (eI4A)-dependent ARF6 and AMAP1 translation by upregulating mTORC1. Mut-TP53 promoted ARF6 activation by PDGF, which in turn increased PDGFR-β expression. The ARF6/AMAP1 pathway was essential for PD-L1 recycling [43]. TP53 also regulates the expression of miR-34a, which can negatively regulate PD-L1 expression and result in immunosuppression and metastasis [45]. An additional mechanism by which mut-TP53 promotes metastasis is the lack of induction of miRs, such as miR-34a, which inhibit the growth and metastasis of PDAC [46].

Nucleolar protein 14 (NOP14) expression is also increased in PDAC tumors. NOP14 promotes cell mobility and invasiveness. NOP14 promotes the stability of mut-TP53, which suppresses p21^Cip−1^ expression at the transcriptional level through the induction of miR-17-5p [47].

Mutant TP53 with GOF activity may be able to promote gene expression that stimulates clonogenicity in the presence of low doses of doxorubicin. It has been shown by others that doxorubicin will support the growth of certain ovarian cancer cells [48]. The TP53 is mutated at a very high frequency in ovarian cancers [49].

The mechanisms of transformation in TP53-null tumors are not so clear or as well studied. One would expect that the induction of p21^Cip−1^ and other negative regulators regulated by TP53, which normally suppress cell cycle progression and metastasis, would be inhibited, but p21^Cip−1^ can be activated by TP53-independent mechanisms [50,51]. Interestingly, certain phospholipid-controlling proteins may be potential targets in the growth of TP53-null cells, namely, phosphatidylinositol 5-phosphate 4-kinase type-2α (PIP4K2α) and PIP4K2β [52].

In our studies, we document the different effects that the APR-246 has in cells which have a GOF TP53 mutation, cells which have a GOF TP53 mutation and an introduced WT-TP53, cells which do not express the TP53 protein, and cells which previously did not express the TP53 protein and now express the WT-TP53. Our studies indicate that the APR-246 can enhance the effects of chemotherapeutic drugs and signal transduction inhibitors in the presence of WT-TP53 or a GOF TP53 mutant in MIA-PaCa-2 cells. In contrast, the APR-246 inhibitor did not enhance the effects of chemotherapeutic drugs and signal transduction inhibitors in cells which did not express TP53, but when WT-TP53 was introduced into these cells, the APR-246 compound increased the effects of the chemotherapeutic drugs and signal transduction inhibitors dramatically.

TP53 is mutated in approximately 70% of esophageal adenocarcinomas, and APR-246 has been demonstrated to synergize with 5FU and other drugs in preclinical models of esophageal cancer [53]. We observed that APR-246 increased the sensitivity of both MIA-PaCa-2 + pLXSN and MIA-PaCa-2 + WT-TP53 to 5FU. The effects of APR-246 were higher in MIA-PaCa-2 + WT-TP53 than MIA-PaCa-2 + pLXSN cells. Interestingly, in the previous study, the researchers also demonstrated that APR-246 did not synergize with 5FU in TP53-null esophageal cancer cells, but when WT-TP53 was introduced into the cells, synergy was observed [53]. We observed that APR-246 increased the sensitivity of PANC-28 + WT-TP53 to 5FU but not PANC-28 + pLXSN.

Additionally, it was demonstrated that APR-246 treatment restored the sensitivity of drug-resistant ovarian cancer to doxorubicin [54,55]. We observed that APR-246 increased the sensitivity of both MIA-PaCa-2 + pLXSN and MIA-PaCa-2 + WT-TP53 to doxorubicin. The effects of APR-246 were higher in MIA-PaCa-2 + WT-TP53 than MIA-PaCa-2 + pLXSN cells. We observed that APR-246 could sensitize PANC-28 + pLXSN cells to doxorubicin, but the effects of APR-246 were much more significant in PANC-28 + WT-TP53 cells. In the studies by Liu et al. [53], they did observe that the effects of anthracycline epirubicin were TP53-independent. Epirubicin is a 4′-epimer of doxorubicin. While the efficacy of epirubicin is similar to doxorubicin, epirubicin has a different toxicity profile, particularly in regard to cardiotoxicity. Epirubicin has replaced doxorubicin in many of the anthracycline-based treatments of breast cancer. Four clinical trials have been performed with pancreatic cancer and epirubicin.

There are clinical trials examining the potential of gilteritinib and APR-246 in treating acute myelogenous leukemia [56]. Since FLT-3 and TP53 are frequently mutated in AML, it is logical to consider targeting both FLT-3 and TP53 in various cancers which have the overexpression of FLT-3 and mutant TP53.

The effects of addition of APR-246 to gilteritinib were much greater in MIA-PaCa-2 + pLXSN than in MIA-PaCa-2 + WT-TP53 cells. Gilteritinib inhibits the AXL, ALK, and FLT3 proteins. The interactions of AXL and TP53 are complicated, as AXL can suppress TP53 expression and TP53 can inhibit AXL expression through the induction of miR-34a. In the presence of the mutant TP53 reactivator APR-246, AXL may be present at lower levels via the TP53/mi34a interaction, which synergizes with the gilteritinib AXL/ALK/FMS inhibitor and results in the suppression of proliferation. Alternatively, the suppression of AXL protein levels in cells containing GOF mut-TP53 may result in increased levels of the GOF mut-TP53 protein, which can alter the activity of many proteins and pathways. Other possibilities also exist to explain this result. In contrast, the effects of APR-246 on the sensitivity to gilteritinib were minimal in PANC-28 + pLXSN cells, which lack TP53 (both GOF mut-TP53 and WT-TP53). Much more significant effects were observed in PANC-28 + WT-TP53 cells.

NCT04419389 is a clinical trial to examine the effects of APR-246 in combination with either the Bruton tyrosine kinase inhibitor acalabrutinib or the BCL2 inhibitor venetoclax for therapy in subjects with non-Hodgkin lymphomas (NHL), including chronic lymphocytic leukemia (CLL) and mantle cell lymphoma (MCL).

There are clinical trials with a BCL2 inhibitor and APR246 in hematopoietic malignancies [57]. Elevated BCL2 expression has been associated with the metastasis of PDAC [58]. We observed significant interactions between the BCL2/BCXL inhibitor ABT-737 in both MIA-PaCa-2 + pLXSN and MIA-PaCa-2 + WT-TP53 cells. In contrast, in PANC-28 + pLXSN cells, there was a mild effect when the cells were treated with APR-246 and ABT-737. In contrast the effects were much more dramatic in PANC-28 + WT-TP53 cells containing the introduced WT-TP53. We observed significant interactions between the BCL2/BCXL inhibitor ABT-737 in both MIA-PaCa-2 + pLXSN and MIA-PaCa-2 + WT-TP53 cells. In contrast, in PANC-28 + pLXSN cells, there was a mild effect when the cells were treated with APR-246 and ABT-737. In contrast, the effects were much more dramatic in PANC-28 + WT-TP53 cells containing introduced WT-TP53.

APR-246 has been, and is being, examined in at least thirteen clinical trials with various cancer types. In some cases, the effects of APR-246 have been examined in combination with azacitidine (in MDS and AML), dabrafenib (in V600 BRAF-mutant melanomas), pembrolizumab (an immunotherapeutic which targets PD-1, also known as Keytruda), and venetoclax [15,59,60,61,62].

In a phase 2 clinical trial with MDS and AML patients with mutant TP53, a combination of APR-246 with azacitidine resulted in higher survival and response rates than treatment with azacitidine by itself. This combination was observed to downregulate signaling through FLT-3 [62], an important growth factor receptor which is often mutated/deregulated in AML. Some phase 3 clinical trials with APR-246 have been performed. One phase 1 clinical trial (NCT04638309) with the structurally related APR-548 developed by the same company (Aprea Therapeutics Boston, MA, USA, and Stockholm, Sweden), that has higher oral bioavailability, is ongoing.

Our studies point to the value of knowing what type of TP53 mutation(s) there is/are in cancer patients that may be treated with APR-246 and next-generation related compounds. These compounds may not have significant effects in cells which have deleted or silenced TP53 expression. While this observation does sound obvious, however, there are other TP53-related molecules, such as TP63 and TP73, which could be activated by the APR-246 compound in PANC-28 + pLXSN cells. In fact, we observed low background effects when PANC-28 + pLXSN cells were treated with APR-246.

The identification of novel reactivators of tumor suppressor genes is a critical basic and clinical research area [63]. Tumor suppressor genes are frequently inactivated in human malignancies. APR-246 and structurally related compounds which function by reactivating mutant tumor suppressor proteins represent important additions to effective cancer therapy. Our studies are the first to determine the effects of TP53 reactivation in combination with these drugs in PDAC cells and to suggest potential interactions which could be further explored in PDAC therapy.

## Figures and Tables

**Table 1:** Effects of WT-TP53 on the sensitivity of MIA-PaCa-2 PDAC cells to APR-246 ^1^.

Drug/Agent	MIA-PaCa-2 + pLXSN (−APR-246)	MIA-PaCa-2 + pLXSN (+12.5 nM APR-246)	Fold Change +/− APR-246	MIA-PaCa-2 + WT-TP53 (−APR-246)	MIA-PaCa-2 + WT-TP53 (+12.5 nM APR-246)	Fold Change +/− APR-246
5FU (nucleoside analogue)	7 µM	1.8 µM	3.9 × ↓	3.2 µM	0.3 µM	10.7 × ↓
Doxorubicin (topoisomerase inh.)	220 nM	60 nM	3.6 × ↓	50 nM	18 nM	2.8 × ↓
Gilteritinib (ALK/AXL/FLT3 inh.)	750 nM	8.5 nM	88.2 × ↓	400 nM	200 nM	2 × ↓
ABT-737 (BCL2/BCLXL inh.)	2000 nM	4 nM	500 × ↓	1000 nM	110 nM	9.1 × ↓

^1^ Determined as described in 30–32.

**Table 2:** Effects of WT-TP53 on the sensitivity of PANC-28 PDAC cells to APR-246 ^1^.

Drug/Agent	PANC-28 + pLXSN (−APR-246)	PANC-28 + pLXSN (+12.5 nM APR-246)	Fold Change +/− APR-246	PANC-28 + WT-TP53 (−APR-246)	PANC-28 + WT-TP53 (+12.5 nM AP-246)	Fold Change +/− APR-246
5FU (nucleoside analogue)	30 µM	30 µM	1 ×	6.5 µM	0.1 µM	65 × ↓
Doxorubicin (topoisomerase inh.)	210 nM	170 nM	1.2 × ↓	100 nM	15 nM	6.7 × ↓
Gilteritinib (ALK/AXL/FLT3 inh.)	900 nM	800 nM	1.1 × ↓	280 nM	3.8 nM	73.7 × ↓
ABT-737 (BCL2/BCLXL inh.)	1000 nM	800 nM	1.3 × ↓	100 nM	2 nM	50 × ↓

^1^ Determined as described in [30,31,32].

## Data Availability

The data presented in this study are available on request from the corresponding authors.
